# Supplementary material for: Gut microbiome remodeling and metabolomic profile improves in response to protein pacing with intermittent fasting versus continuous caloric restriction
Source: Nat Commun. 2024 May 28;15:4155. doi: 10.1038/s41467-024-48355-5 (PMC11133430; doi:10.1038/s41467-024-48355-5)
Supplement: Supplementary file 1 — Supplementary Information [file 41467_2024_48355_MOESM1_ESM.pdf]

Supplemental Information

Gut microbiome remodeling and metabolomic profile improves in response to intermittent fasting with protein pacing versus continuous caloric restriction

Alex E. Mohr[1,2], Karen L. Sweazea[1,2,3], Devin A. Bowes[2], Paniz Jasbi[4,5], Corrie M. Whisner[1,2], Dorothy D. Sears[1], Rosa Krajmalnik-Brown[2], Yan Jin[6], Haiwei Gu[1,6], Judith Klein-Seetharaman[1,4], Karen M. Arciero[7], Eric Gumpricht[8], Paul J. Arciero[7,9]*

[1]College of Health Solutions, Arizona State University, Phoenix, AZ, USA

[2]Biodesign Institute Center for Health Through Microbiomes, Arizona State University, Tempe, AZ, USA

[3]Center for Evolution and Medicine, College of Liberal Arts and Sciences, Arizona State University, Tempe, AZ, USA

[4]School of Molecular Sciences, Arizona State University, Tempe, AZ, USA

[5]Systems Precision Engineering and Advanced Research (SPEAR), Theriome Inc., Phoenix, AZ, USA

[6]Center of Translational Science, Florida International Univeristy, Port St. Lucie, FL, USA

[7]Human Nutrition and Metabolism Laboratory, Department of Health and Human Physiological Sciences, Skidmore College, Saratoga Springs, NY, USA

[8]Isagenix International, LLC, Gilbert, AZ, USA

[9]School of Health and Rehabilitation Sciences, Department of Sports Medicine and Nutrition, University of Pittsburgh, Pittsburgh, PA, USA

***Corresponding Author: parciero@skidmore.edu**

Page S-4, Table S1. Dietary regimens of the intermittent fasting protein pacing (IF-P) and continuous calorie restriction (CR) diet groups.

Page S-5, Table S2. Nested permutational analysis of variance model parameters and term results for Bray-Curtis dissimilarity matrix of diet groups, intermittent fasting protein pacing (IF-P) and continuous calorie restriction (CR) and time.

Page S-6, Table S3. Plasma cytokine concentrations between intermittent fasting protein pacing (IF-P) and continuous calorie restriction (CR) diet groups at baseline and weeks four and eight.

Page S-7, Table S4. Significance, fold change (FC), and area under the curve (AUC) details of metabolite markers of intermittent fasting protein pacing (IF-P) and continuous calorie restriction (CR).

Page S-8, Table S5. Variance and difference between intermittent fasting protein pacing (IF-P) and continuous calorie restriction (CR) for the eight latent factors extracted from multi-omics factor analysis.

Page S-9, Table S6. Baseline characteristics of high and low response subgroup study participants.

Page S-10, Table S7. Permutational analysis of variance model parameters and term results for Bray-Curtis dissimilarity matrix of high and low response group and time.

Page S-11, Fig S1. (**a**) CONSORT diagram describing enrollment, allocation, and data analysis of the parent clinical study. (**b**) Daily consumption of macronutrients measured by mass (g) at baseline, week four, and week eight between the intermittent fasting protein pacing (IF-P) and continuous calorie restriction (CR) groups. There were no differences between groups at baseline for all dietary intake variables (two-sided Student's t-test $p < 0.05$). (**c**) Targeted gas chromatography-mass spectrometry (GC-MS) measurement of fecal short-chain fatty acids (SCFAs) over the study duration. No significant effects were observed for time or interaction (linear mixed effects models, two-sided $p > 0.05$). For all panels, IF-P: *n* = 20, CR: *n* = 19. Source data are provided as a Source Data file.

Page S-12, Fig S2. Cytokines and the microbiome in intermittent fasting protein pacing (IF-P) participants. (**a**) Grid-fused least absolute shrinkage and selection operator (GFLASSO) regression identified several genera with correlative relationships with our cytokine panel. Two-sided spearman correlations of (**b**) IL-4 and (**c**) IL-13 with microbial genera using intervention samples (weeks four and eight). For all panels, IF-P: *n* = 20, CR: *n* = 19. Source data are provided as a Source Data file.

Page S-13, Fig S3. Analysis of plasma metabolome. (**a**) As assessed by the Canberra distance metric, no differences between intermittent fasting protein pacing (IF-P) and continuous calorie restriction (CR) were detected over the intervention in the metabolome (two-sided Wilcoxon rank sum test). (**b**) Five metabolites with a VIP > 1.0 (2,3-dihydroxybenzoic acid, malonic acid, protocatechuic acid, agmatine, and myo-inositol) were retained to construct an enhanced orthogonal projection to latent structures discriminant analysis (OPLS-DA) model. In contrast, model fit was assessed with 100-fold leave-one-out cross-validation (LOOCV). (**c**) A refined OPLS-DA model overview showing predictive and explanatory capacity ($Q^2 = 0.460$, $p < 0.001$; $R^2 = 0.506$, $p < 0.001$). (**d**) Receiver operating characteristic analysis of the OPLS-DA model (AUC = 0.929, 95% CI: 0.868-0.973, sensitivity = 0.8, specificity = 0.9). For all panels, IF-P: *n* = 20, CR: *n* = 19. Source data are provided as a Source Data file.

Page S-14, Fig. S4. (**a**) Short-chain fatty acid (SCFA) analysis from high and low weight loss responders did not differ significantly (two-sided Wilcoxon rank-sum test, p.adj ≥ 0.10). Values from the four principle SCFAs generated from targeted gas chromatography-mass spectrometry (GC-MS) are displayed as log2

fold change (FC). (**b**) Change in the fecal metabolome was not significantly different between weight loss response classification when comparing log2FC values (two-sided Wilcoxon rank-sum test, p.adj > 0.10). For visual interpretability, changes are displayed at the chemical sub-class level. For all panels, High: *n* = 5, Low: *n* = 5. Source data are provided as a Source Data file.

Page S-15, Fig. S5. (**a**) Bray Curtis dissimilarity of functional pathways derived from metagenomic analysis with (**b**) top PERMANOVA model coefficients (analysis: pathway ~ time). (**c**) Top PERMANOVA model coefficients (analysis: pathway ~ time) from fecal metabolome, including the subclass, amino acids, peptides, and analoges. (**d**) Alluvial plot displaying the variation in abundance of the most abundant chemical subclasses (>1% relative abundance) over time. For visual clarity, the less abundant metabolite classes (<1% relative abundance) are not displayed. (**e**) Heatmap of the fecal metabolites captured by pathway analysis from the case study assessment. Source data are provided as a Source Data file.

Table S1. Dietary regimens of the intermittent fasting protein pacing (IF-P) and continuous calorie restriction (CR) diet groups.

Variable	IF-P		CR	
	**Men (1800 kcals/day)**	**Women (1450 kcals/day)**	**Men (1500 kcals/day)**	**Women (1200 kcals/day)**
Breakfast (0600–0800)	Liquid meal replacement shake; 400-450 kcals, **36 g protein**, 40-43 g carbohydrate, 12-15 g fat; Caffeine beverage; Antioxidant & Adaptogen mix; 20 kcals	Liquid meal replacement shake; 350-400 kcals, **30 g protein**, 35-40 g carbohydrate, 10-12 g fat; Caffeine beverage; Antioxidant & Adaptogen mix; 20 kcals	Oatmeal, fruit, milk, honey, egg, whole grain toast, coffee; 400 kcals, **20 g protein**, 55 g carbohydrate, 11 g fat	Oatmeal, fruit, milk, honey, egg, coffee; 350 kcals, **15 g protein**, 50 g carbohydrate, 10 g fat
Lunch (1100–1300)	Choice of liquid meal replacement shake or Fresh vegetables with choice of fish/poultry/ beef/plant-based protein; 400 kcals, **36 g protein**, 40 g carbohydrate, 12 g fat	Choice of liquid meal replacement shake or Fresh vegetables with choice of fish/poultry/ beef/plant-based protein; 350 kcals, **30 g protein**, 35 g carbohydrate, 10 g fat	Whole grain pita, poultry/fish/beef/plant-based protein, tomato, lettuce, mayonnaise, mustard, apple, skim milk; 400 kcals, **20 g protein**, 60 g carbohydrate, 10 g fat	Whole grain pita, poultry/fish/beef/plant-based protein, tomato, lettuce, mayonnaise, mustard, apple, skim milk; 350 kcals, **15 g protein**, 50 carbohydrate, 10 g fat
Mid-Afternoon snack (1400–1600)	Greek yogurt, fruit; 200 kcals, **20 g protein,** 15 g carbohydrate, 5 g fat		Yogurt, fruit; 200 kcals, **10 g protein,** 30 g carbohydrate, 5 g fat	
Dinner (1700–1900)	Fish/Poultry/Beef, fresh vegetables, chopped nuts, dried fruit, olive oil, milk; 500 kcals, **36 g protein,** 50 g carbohydrate, 18 g fat	Fish/Poultry/Beef, fresh vegetables, chopped nuts, dried fruit, olive oil, milk; 450 kcals, **30 g protein**; 50 g carbohydrate; 15 g fat	Whole grain, fish/beef/ poultry/plant-based protein, vegetables, nuts, dried fruit, olive oil, milk; 500 kcals, **25 g protein**, 80 g carbohydrate, 12 g fat	Whole grain, fish/beef/ poultry/plant-based protein, vegetables, nuts, dried fruit, olive oil, milk; 450 kcals, **20 g protein,** 75 g carbohydrate, 8 g fat
Evening snack (2100–2200)	Protein shake/bar snack; 250 kcals, **30 g protein,** 20 g carbohydrate, 8 g fat	Protein shake/bar snack; 225 kcals, **25 g protein**; 20 g carbohydrate; 8 g fat	Milk, crackers; 225 kcals, **15 g protein**, 30 g carbohydrate, 4 g fat	Milk, crackers; 225 kcals, **10 g protein**, 30 g carbohydrate, 5 g fat

Intermittent Fasting Day Intake for IF-P

Antioxidant plant-based powder	4/day	160 kcal total
Plant-based herbal adaptogen powder	2/day	40 kcal total
Collagen bone broth	2/day	45 kcal total
Low-glycemic protein crackers	1/day	100 kcal total
Electrolyte beverage	1/day	20 kcal total
Optional foods:	1/day	Dark chocolate square; antioxidant/caffeine beverage; ½ nut bar; fresh vegetable/fruit, nut/seed mix (all < 50 kcal total for IF1-P and 150-200 kcal total for IF2-P)

Table S2. Nested permutational analysis of variance model parameters and term results for Bray-Curtis dissimilarity matrix of diet groups, intermittent fasting protein pacing (IF-P) and continuous calorie restriction (CR) and time.

	DF	SS	R^2	F	p
**Participant**	38	22.664	0.749	7.056	0.001
**Time*Group**	2	0.542	0.018	3.206	0.001
**Time*Group (nested: participant)**	37	3.724	0.123	1.191	0.003
**Residuals**	39	3.297	0.109		
**Total**	116	30.227	1.000		

Abbreviations: DF, degrees of freedom; SS, sum of squares.

Table S3. Plasma cytokine concentrations between intermittent fasting protein pacing (IF-P) and continuous calorie restriction (CR) diet groups at baseline and weeks four and eight.

**Variable**	**Baseline**		**Week 4**		**Week 8**	
	**IF-P (*n* = 20)**	**CR (*n* = 19)**	**IF-P (*n* = 20)**	**CR (*n* = 19)**	**IF-P (*n* = 20)**	**CR (*n* = 19)**
GM-CSF (pg/mL)	80.34±13.93	85.81±26.56	82.23±13.92	84.21±26.95	81.14±12.79	77.35±22.46
IFNγ (pg/mL)	8.04±0.77	9.56±1.13	8.64±1.03	9.51±1.04	7.64±0.65	9.41±0.87
IL-1β (pg/mL)	4.05±0.36	4.21±0.49	4.11±0.35	4.16±0.43	4.02±0.30	3.96±0.41
IL-2 (pg/mL)	6.04±0.55	7.18±0.92	6.31±0.66	6.67±0.79	5.45±0.43	6.67±0.73
IL-4 (pg/mL)	53.15±14.59	73.26±22.05	72.22±17.27[a]	84.19±24.07	79.64±16.92[a]	76.96±20.81
IL-5 (pg/mL)	5.72±3.66	2.41±0.29	5.85±3.63	2.33±0.32	6.05±3.84	2.21±0.24
IL-6 (pg/mL)	3.99±0.98	5.33±1.24	4.86±1.12[a]	5.21±1.30	5.43±1.08[a]	4.96±1.23
IL-8 (pg/mL)	14.84±4.63	14.88±3.51	18.72±5.22[a]	15.47±3.54	20.80±5.04[a]	13.55±3.09
IL-10 (pg/mL)	14.79±1.76	17.85±2.73	17.03±2.07	19.09±2.59	17.80±2.20	18.57±2.73
IL-12p70 (pg/mL)	7.31±0.59	9.56±1.01	6.82±0.53	8.67±0.93	6.42±0.49	9.16±1.01
IL-13 (pg/mL)	14.83±2.80	17.17±2.84	18.06±3.14[a]	17.08±2.91	17.94±2.87[a]	16.68±3.21
IL-17A (pg/mL)	18.51±1.25	19.44±2.23	17.73±1.36	18.47±1.99	16.69±1.19	18.55±1.88
IL-23 (pg/mL)	1442.30±276.38	1491.48±210.08	1379.28±265.49	1502.74±217.47	1347.88±264.22	1443.68±194.26
TNFα (pg/mL)	8.28±0.73	9.94±0.70	8.81±0.70	9.62±0.74	8.31±0.58	9.47±0.65

Data are reported as mean ± SEM. Statistical analysis was conducted using linear mixed effect modeling. All p-values were calculated using two-sided tests. Significant increases compared to baseline values: [a]p.adj < 0.1

Table S4. Significance, log fold-change (logFC), and area under the curve (AUC) details of metabolite markers of intermittent fasting protein pacing (IF-P) and continuous calorie restriction (CR).

Metabolite	p*	p.adj*	logFC**	AUC
2,3-Dihydroxybenzoic acid	1.6e-07	2.2e-05	-0.519	0.816
Malonic acid	3.3e-06	2.3e-04	0.464	0.808
Choline	1.6e-05	7.2e-04	-0.249	0.819
Agmatine	6.9e-05	0.002	-0.278	0.799
Protocatechuic acid	1.9e-04	0.005	-0.334	0.738
Myo-inositol	0.002	0.036	-0.309	0.718
Dulcitol	0.002	0.037	-0.305	0.630
Oxaloacetic acid	0.002	0.037	-0.213	0.760
Xylitol	0.002	0.037	-0.187	0.722
N-Acetylglutamine	0.003	0.037	-0.220	0.669
Asparagine	0.003	0.037	-0.171	0.729
Sorbitol	0.005	0.063	-0.271	0.606
Cytidine	0.006	0.069	-0.206	0.662
Acetylcarnitine	0.008	0.081	0.185	0.582
Urate	0.009	0.089	-0.138	0.731
Kynurenine	0.015	0.119	-0.171	0.639
9-Octadecynoic acid	0.015	0.119	0.1563	0.651
Decanoylcarnitine	0.021	0.150	-0.201	0.589
Pyroglutamic acid	0.024	0.150	-0.164	0.644
5-Hydroxyindoleacetic acid	0.024	0.150	-0.158	0.642
Tryptophan	0.022	0.150	-0.139	0.595
Asymmetric dimethylarginine	0.021	0.150	-0.119	0.629
D-Mannitol	0.026	0.159	-0.287	0.613
L-(+)-Arabinose	0.031	0.179	-0.199	0.618
D-Galacturonic acid	0.033	0.185	-0.168	0.615
2-Hydroxyglutarate	0.036	0.186	-0.152	0.611
Dimethylglycine	0.036	0.187	-0.145	0.623
Alanine	0.037	0.187	-0.114	0.639
Sarcosine	0.038	0.187	-0.113	0.631
Indole-3-lactic acid	0.049	0.210	-0.122	0.599
Palmitic acid	0.049	0.210	0.120	0.608
Valeric acid	0.049	0.210	0.126	0.596

*Derived from age-, sex-, and time-adjusted general linear model (GLM) with Benjamini–Hochberg multiple testing correction. All p-values were calculated using two-sided tests.
**Analyzed as IF-P/CR.

Table S5. Variance and difference between intermittent fasting protein pacing (IF-P) and continuous calorie restriction (CR) for the eight latent factors extracted from multi-omics factor analysis.

Factor	R^{2*}	p*	p.adj*
Factor 1	11.98	4e-05	3.2e-04
Factor 2	8.78	0.61	0.697
Factor 3	7.71	0.063	0.169
Factor 4	6.92	0.830	0.830
Factor 5	6.71	0.093	0.186
Factor 6	5.28	0.002	0.007
Factor 7	5.26	0.150	0.240
Factor 8	4.27	0.510	0.679

*Two-sided Wilcoxon rank sum test performed between IF-P and CR groups for each factor.

Table S6. Baseline characteristics of subgroup study participants.

**Variable**	**"Low" (*n*=5)**	**"High" (*n*=5)**	**p***
Age, mean ± SD	50.8 ± 2.3	47.2 ± 8.9	0.249
Sex, % (n)			
Men	40 (2)	40 (2)	
Women	60 (3)	60 (3)	
Race/ethnicity, % (n)			
White	100 (5)	80 (4)	
Asian	0 (0)	20 (1)	
Height (cm)	168.1 ± 16.8	175.1 ± 4.9	0.602
Weight (kg)	81.9 ± 18.1	108.9 ± 30.8	0.117
Body fat (%)	36.3 ± 6.4	41.6 ± 7.6	0.251
Body mass index (kg/m2)	28.8 ± 2.8	35.6 ± 10.2	0.117
Waist circumference (cm)	96.0 ± 11.3	115.3 ± 21.0	0.076
Physical Activity (kcals/day)	366 ± 357	371 ± 214	0.754
Diet, mean ± SD			
Kcals	2536 ± 595	2640 ± 556	0.754
Carbohydrates (g)	252.5 ± 54.4	267.9 ± 74.2	0.917
Sugar (g)	76.1 ± 40.7	107.9 ± 46.9	0.347
Fiber (g)	19.7 ± 3.9	21.8 ± 12.5	0.602
Protein (g)	108.4 ± 22.9	103.2 ± 40.6	0.251
Fat (g)	102.9 ± 35.0	123.3 ± 25.9	0.175
Sodium (mg)	3696.9 ± 759.9	3704.7 ± 1675.5	0.917

*Two-sided Mann-Whitney U test, alpha = 0.05.

Table S7. Permutational analysis of variance model parameters and term results for Bray-Curtis dissimilarity matrix of high and low response group and time.

	DF	SS	R^2	F	p
**Participant**	9	3.237	0.711	3.615	0.001
**Time*Group**	2	0.519	0.114	2.609	0.001
**Residuals**	8	0.796	0.175		
**Total**	19	4.553	1.000		

Abbreviations: DF, degrees of freedom; SS, sum of squares.

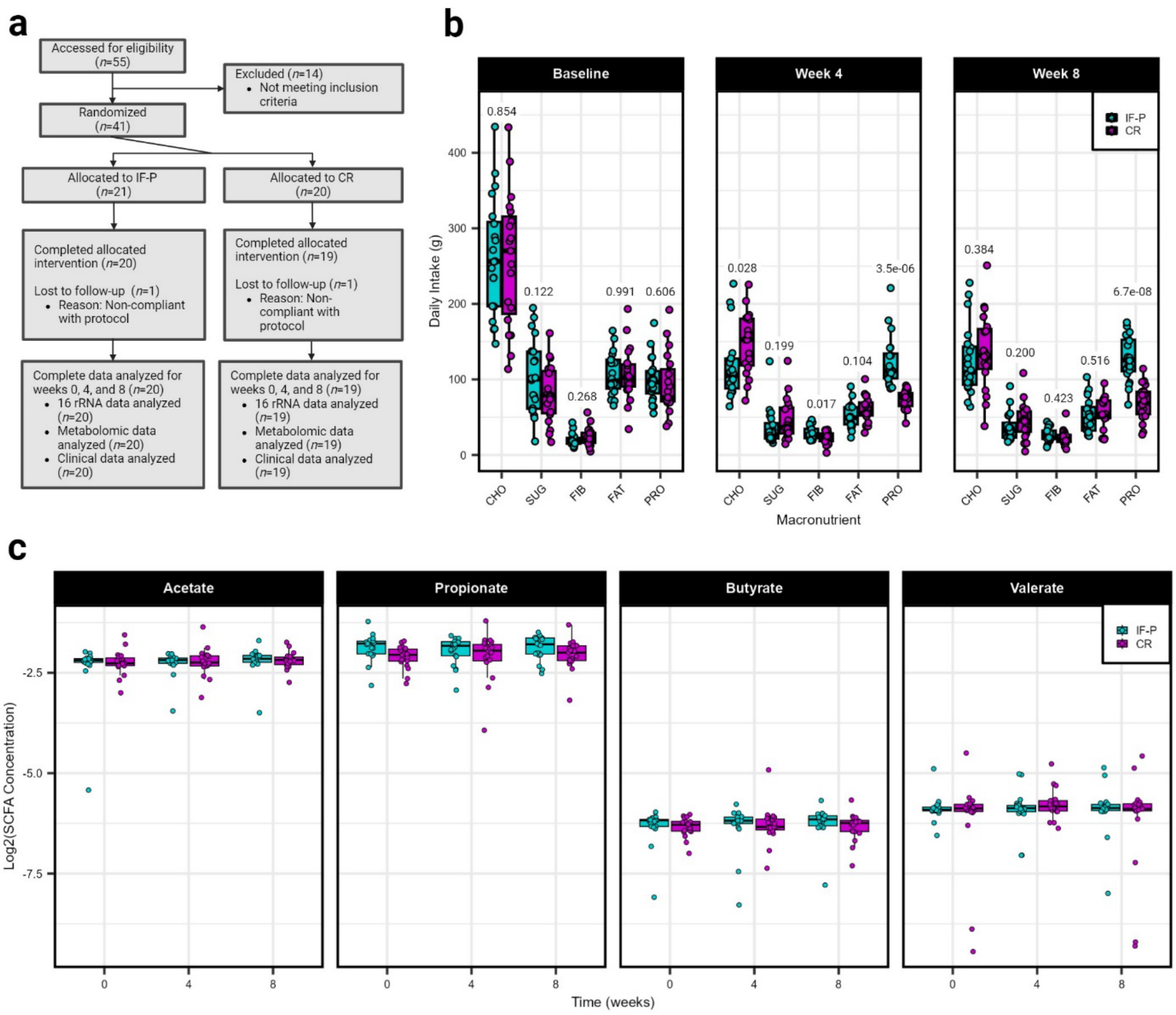

Fig S1. (**a**) CONSORT diagram describing enrollment, allocation, and data analysis of the parent clinical study. (**b**) Daily consumption of macronutrients measured by mass (g) at baseline, week four, and week eight between the intermittent fasting protein pacing (IF-P) and continuous calorie restriction (CR) groups. There were no differences between groups at baseline for all dietary intake variables (two-sided Student's t-test $p < 0.05$). (**c**) Targeted gas chromatography-mass spectrometry (GC-MS) measurement of fecal short-chain fatty acids (SCFAs) over the study duration. No significant effects were observed for time or interaction (linear mixed effects models, two-sided $p > 0.05$). For all panels, IF-P: n = 20, CR: n = 19. Source data are provided as a Source Data file.

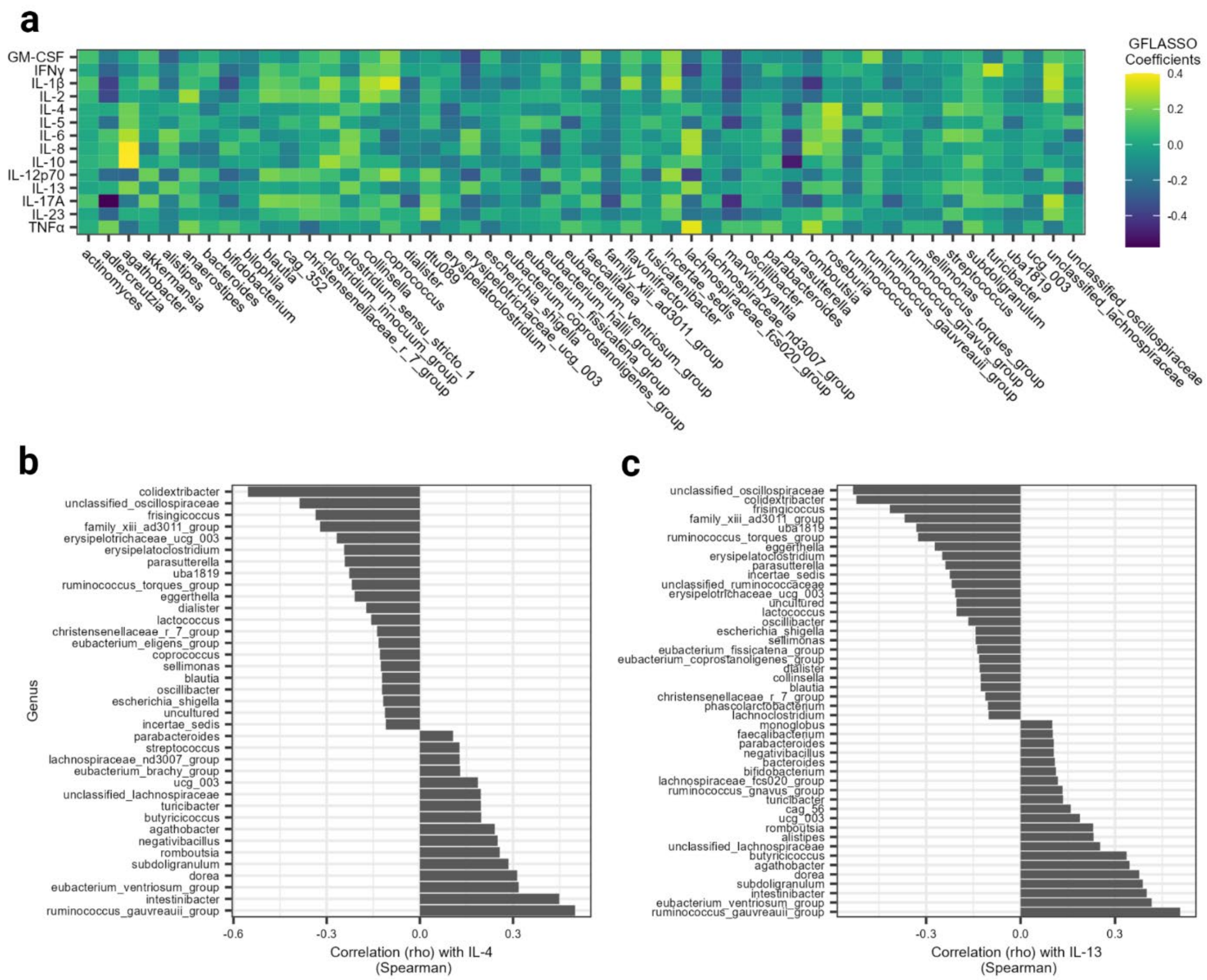

Fig S2. Cytokines and the microbiome in intermittent fasting protein pacing (IF-P) participants. (**a**) Grid-fused least absolute shrinkage and selection operator (GFLASSO) regression identified several genera with correlative relationships with our cytokine panel. Two-sided spearman correlations of (**b**) IL-4 and (**c**) IL-13 with microbial genera using intervention samples (weeks four and eight). For all panels, IF-P: *n* = 20, CR: *n* = 19. Source data are provided as a Source Data file.

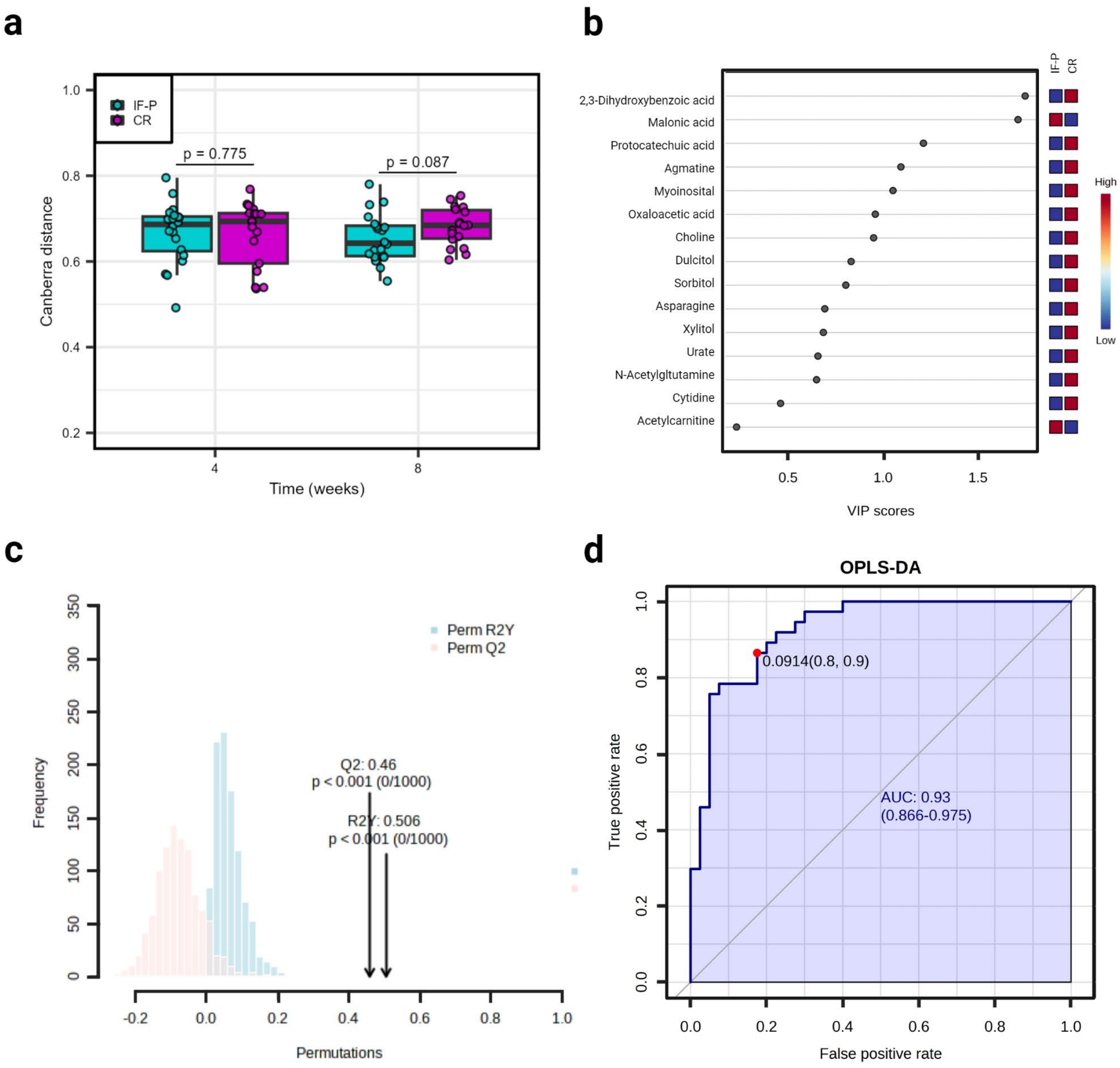

Fig S3. Analysis of plasma metabolome. (**a**) As assessed by the Canberra distance metric, no differences between intermittent fasting protein pacing (IF-P) and continuous calorie restriction (CR) were detected over the intervention in the metabolome (two-sided Wilcoxon rank sum test). (**b**) Five metabolites with a VIP > 1.0 (2,3-dihydroxybenzoic acid, malonic acid, protocatechuic acid, agmatine, and myo-inositol) were retained to construct an enhanced orthogonal projection to latent structures discriminant analysis (OPLS-DA) model. In contrast, model fit was assessed with 100-fold leave-one-out cross-validation (LOOCV). (**c**) A refined OPLS-DA model overview showing predictive and explanatory capacity (Q^2 = 0.460, $p < 0.001$; R^2 = 0.506, $p < 0.001$). (**d**) Receiver operating characteristic analysis of the OPLS-DA model (AUC = 0.929, 95% CI: 0.868-0.973, sensitivity = 0.8, specificity = 0.9). For all panels, IF-P: *n* = 20, CR: *n* = 19. Source data are provided as a Source Data file.

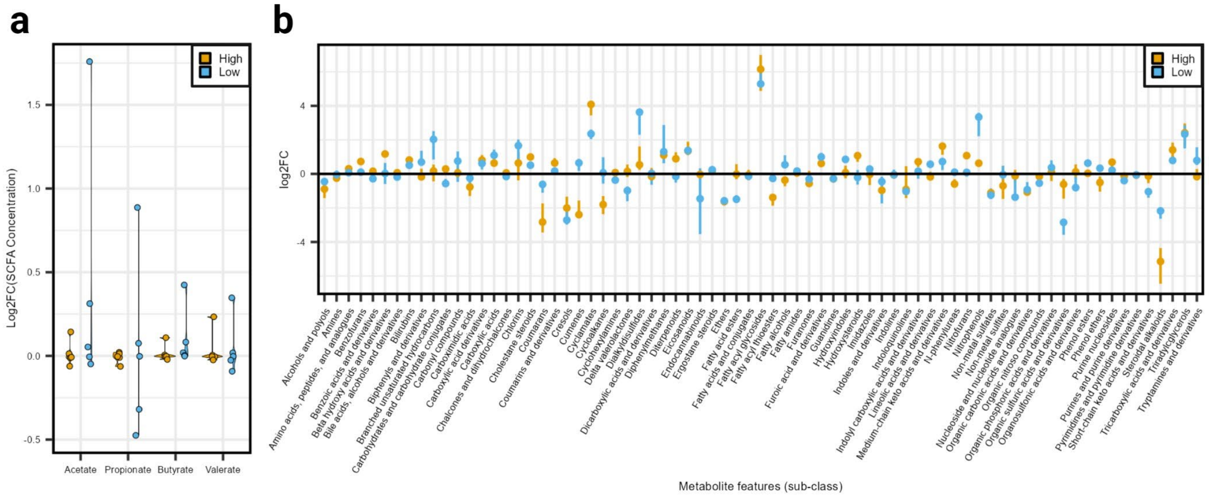

Fig. S4. (**a**) Short-chain fatty acid (SCFA) analysis from high and low weight loss responders did not differ significantly (two-sided Wilcoxon rank-sum test, p.adj ≥ 0.10). Values from the four principle SCFAs generated from targeted gas chromatography-mass spectrometry (GC-MS) are displayed as log2 fold change (FC). (**b**) Change in the fecal metabolome was not significantly different between weight loss response classification when comparing log2FC values (two-sided Wilcoxon rank-sum test, p.adj > 0.10). For visual interpretability, changes are displayed at the chemical sub-class level. For all panels, High: *n* = 5, Low: *n* = 5. Source data are provided as a Source Data file.

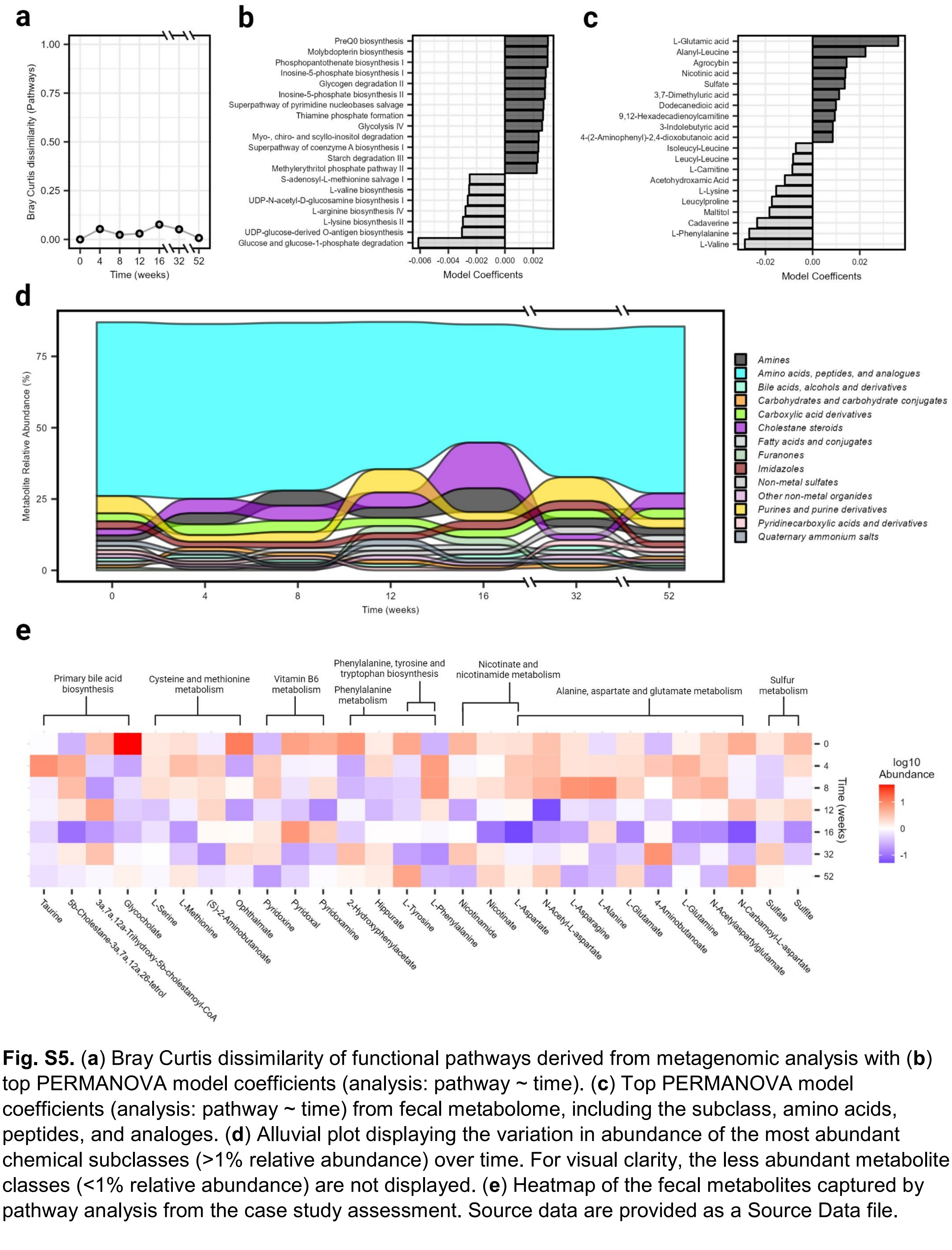

Fig. S5. (**a**) Bray Curtis dissimilarity of functional pathways derived from metagenomic analysis with (**b**) top PERMANOVA model coefficients (analysis: pathway ~ time). (**c**) Top PERMANOVA model coefficients (analysis: pathway ~ time) from fecal metabolome, including the subclass, amino acids, peptides, and analoges. (**d**) Alluvial plot displaying the variation in abundance of the most abundant chemical subclasses (>1% relative abundance) over time. For visual clarity, the less abundant metabolite classes (<1% relative abundance) are not displayed. (**e**) Heatmap of the fecal metabolites captured by pathway analysis from the case study assessment. Source data are provided as a Source Data file.
