# Supplementary material for: Gut microbiome remodeling and metabolomic profile improves in response to protein pacing with intermittent fasting versus continuous caloric restriction
Source: Nat Commun. 2024 May 28;15:4155. doi: 10.1038/s41467-024-48355-5 (PMC11133430; doi:10.1038/s41467-024-48355-5)
Supplement: Supplementary file 3 — Description of Additional Supplementary Files [file 41467_2024_48355_MOESM3_ESM.pdf]

Supplemental Data Legends

NCOMMS-23-43398C: ***Gut microbiome remodeling and metabolomic profile improves in response to protein pacing with intermittent fasting versus continuous caloric restriction***

Supplemental Data 1. Body weight (BW), percent body weight loss from baseline (%BW), adjusted kcal intake, and daily consumption of macronutrients measured by mass (g) for IF-P and CR participants at baseline and weeks 4 and 8.

Supplemental Data 2. 16S rRNA gene copies per gram wet weight, observed amplicon sequence variants (Obs. ASVs), phylogenetic diversity (PD), and intra-individual change in Bray-Curtis dissimilarity (ΔBC) calculated from microbiome samples from IF-P and CR participants at baseline and weeks 4 and 8.

Supplemental Data 3. The percent relative abundance of 28 bacterial families detected in filtered microbiome samples from IF-P and CR participants at baseline and weeks 4 and 8.

Supplemental Data 4. The percent relative abundance of 69 bacterial genera detected in filtered microbiome samples from IF-P and CR participants at baseline and weeks 4 and 8.

Supplemental Data 5. Targeted gas chromatography-mass spectrometry (GC-MS) measurement of fecal short-chain fatty acids (uM) over the study duration from IF-P and CR participants at baseline and weeks 4 and 8.

Supplemental Data 6. Significant (by interaction of group x time) plasma cytokines (IL-4, IL-6, IL-8, and IL-13) concentrations (pg/mL) over the study duration from IF-P and CR participants at baseline and weeks 4 and 8.

Supplemental Data 7. Spearman rho correlation matrix between change (post - pre) in plasma inflammatory cytokines anthropometric and select plasma analytes in the IF-P group.

Supplemental Data 8. The percent relative abundance of 213 bacterial species detected in filtered microbiome samples from "High" and "Low" weight loss responders in the IF-P group at baseline and week 8.

Supplemental Data 9. Log2 fold-change calculated from targeted gas chromatography-mass spectrometry (GC-MS) measurement of fecal short-chain fatty acids (SCFAs) over the study duration for high and low weight loss responders in the IF-P group at baseline and week 8.

Supplemental Data 10. The percent relative abundance of 275 estimated metabolic pathways detected in filtered microbiome samples from "High" and "Low" weight loss responders in the IF-P group at baseline and week 8. Pathway abundance displayed in descending order.

Supplementary Data 11. "High" weight loss response subgroup fecal metabolomic pathway analysis results.

Supplementary Data 12. "Low" weight loss response subgroup fecal metabolomic pathway analysis results.

Supplementary Data 13. The percent relative abundance of the case study participant's gut microbiome at the species-level.

Supplementary Data 14. Estimated metabolic pathways profiled in the case study participant's gut microbiome. Pathway abundance displayed in descending order by percentage over the 52-week period.

Supplementary Data 15. Case study fecal metabolomic pathway analysis results.
