# Supplementary material for: Gut microbiome remodeling and metabolomic profile improves in response to protein pacing with intermittent fasting versus continuous caloric restriction
Source: Nat Commun. 2024 May 28;15:4155. doi: 10.1038/s41467-024-48355-5 (PMC11133430; doi:10.1038/s41467-024-48355-5)
Supplement: Supplementary file 19 — Reporting Summary [file 41467_2024_48355_MOESM19_ESM.pdf]

Corresponding author(s): Paul J. Arciero

Last updated by author(s): Mar 17, 2024

Reporting Summary

Nature Portfolio wishes to improve the reproducibility of the work that we publish. This form provides structure for consistency and transparency in reporting. For further information on Nature Portfolio policies, see our Editorial Policies and the Editorial Policy Checklist.

Statistics

For all statistical analyses, confirm that the following items are present in the figure legend, table legend, main text, or Methods section.

n/a	Confirmed	
☐	☒	The exact sample size (*n*) for each experimental group/condition, given as a discrete number and unit of measurement
☐	☒	A statement on whether measurements were taken from distinct samples or whether the same sample was measured repeatedly
☐	☒	The statistical test(s) used AND whether they are one- or two-sided<br>*Only common tests should be described solely by name; describe more complex techniques in the Methods section.*
☐	☒	A description of all covariates tested
☐	☒	A description of any assumptions or corrections, such as tests of normality and adjustment for multiple comparisons
☐	☒	A full description of the statistical parameters including central tendency (e.g. means) or other basic estimates (e.g. regression coefficient) AND variation (e.g. standard deviation) or associated estimates of uncertainty (e.g. confidence intervals)
☐	☒	For null hypothesis testing, the test statistic (e.g. *F*, *t*, *r*) with confidence intervals, effect sizes, degrees of freedom and *P* value noted<br>*Give P values as exact values whenever suitable.*
☐	☒	For Bayesian analysis, information on the choice of priors and Markov chain Monte Carlo settings
☐	☒	For hierarchical and complex designs, identification of the appropriate level for tests and full reporting of outcomes
☐	☒	Estimates of effect sizes (e.g. Cohen's *d*, Pearson's *r*), indicating how they were calculated

Our web collection on statistics for biologists contains articles on many of the points above.

Software and code

Policy information about availability of computer code

Data collection	No commercial, open source, or custom code used data collection for this study
Data analysis	Statistical analyses were performed using open-source software, R (v4.2.2). The analytical packages utilized for the analysis with versions included: MOFA2 (v1.12.1); GFLASSO (v0.0.0.9000); phyloseq (v1.42.0); nLME (v3.1.160); vegan (v2.6.2); MaAsLin2 (v1.12.0); MetaboAnalystR (v5.0); and ggplot2 (v3.4.1). Other tools included: QIIME2 (v2021.8), FastQC (v0.12.0); TrimGalore (0.6.5); Bowtie2 (2.4.3); MetaPhlAn4 (4.0.2); and HUMAnN 3.0 (v3.7). All code used in this publication can be found at: https://github.com/Alex-E-Mohr/GM-Remodeling-IF-ProteinPacing-vs-CaloricRestriction

For manuscripts utilizing custom algorithms or software that are central to the research but not yet described in published literature, software must be made available to editors and reviewers. We strongly encourage code deposition in a community repository (e.g. GitHub). See the Nature Portfolio guidelines for submitting code & software for further information.

Data

Policy information about availability of data

All manuscripts must include a data availability statement. This statement should provide the following information, where applicable:

- Accession codes, unique identifiers, or web links for publicly available datasets
- A description of any restrictions on data availability
- For clinical datasets or third party data, please ensure that the statement adheres to our policy

The individual de-identified participant microbiome sequencing data can be accessed from the BioProject Database of National Centre for Biotechnology Information with the dataset accession number PRJNA847971. The R code used for analysis and figure generation for reproducibility purposes are available at: https://github.com/Alex-E-Mohr/GM-Remodeling-IF-ProteinPacing-vs-CaloricRestriction. Metadata linking the microbiome sequences with the appropriate sample ID and intervention can be found in Supplemental Data 1.

Research involving human participants, their data, or biological material

Policy information about studies with human participants or human data. See also policy information about sex, gender (identity/presentation), and sexual orientation and race, ethnicity and racism.

Reporting on sex and gender	We report on sex in Table S6.
Reporting on race, ethnicity, or other socially relevant groupings	We report on race and ethnicity in Table S6.
Population characteristics	We describe the covariates throughout the manuscript at lines 294-95, 1022-24; 1033-35; 1046-48
Recruitment	This study included 200 individuals from the Saratoga Springs, New York, area who expressed interest. Potential participants responded to flyers, local newspapers, or emails advertising the study. The number of participants initially screened was 55, of which 41 were eligible for participation. Participants were healthy nonsmoking men and women with overweight and obesity.
Ethics oversight	The study was approved by the Human Subjects Institutional Review Board of Skidmore College (IRB#: 1911-859). All experimental procedures were performed in adherence with related New York state regulations and the Federal Wide Assurance, consistent with the National Commission for the Protection of Human Subjects of Biomedical and Behavioral Research, and in agreement with the Helsinki Declaration (revised in 1983). This trial was registered on March 6, 2020, at ClinicalTrials.gov as NCT04327141.

Note that full information on the approval of the study protocol must also be provided in the manuscript.

Field-specific reporting

Please select the one below that is the best fit for your research. If you are not sure, read the appropriate sections before making your selection.

☒ Life sciences ☐ Behavioural & social sciences ☐ Ecological, evolutionary & environmental sciences

For a reference copy of the document with all sections, see nature.com/documents/nr-reporting-summary-flat.pdf

Life sciences study design

All studies must disclose on these points even when the disclosure is negative.

Sample size	Sample size power analysis was based on body weight/composition to achieve an effect size of F = 0.21 with 80% power at α = 0.05 based on previous data from our lab [see reference 15]. This analysis determined n = 38 total sample size was required to detect a significant mean difference of 1.4-kg weight loss between diet intervention groups (IF-P vs. CR). Thus, our participant size was acceptable. However, we have acknowledged this limitation and how it could influence the results (lines: 707-712).
Data exclusions	The overall compliance rate in each group was high (>90%), defined as consuming more than 90% of the respective meals/supplemented feedings. One participant from each group (IF-P and CR) was noncompliant with the dietary guidelines and was dropped from the analysis. Therefore, descriptive baseline characteristics of the 39 participants (26 women and 13 men) who completed are reported
Replication	Our research incorporated a randomized controlled trial (RCT) design, a hallmark in enhancing the robustness of multi-omic analyses. Furthermore, our study employed repeated measures of primary endpoints and precise methodologies, underpinned by weekly participant contact, strict dietary control, and registered dietitian supervision. These measures collectively contributed to the excellent internal validity of our study. While acknowledging the inherent variability of the gut microbiome among individuals, we are confident that the strength of our design and methodology would yield similar results if replicated exactly by other researchers.<br>In our commitment to transparency and facilitating replication, we've taken several steps. Firstly, our research paper features a comprehensive methodology section, elucidating step-by-step procedures, materials, and any specialized equipment or software used. This level of detail empowers fellow researchers to replicate our methodology with precision. Additionally, we've made all raw data, including

	datasets, code, and analysis scripts, accessible through a publicly available repository, in compliance with ethical and legal standards. This accessibility ensures that others can reproduce our findings using the same data. Moreover, we conducted our data analysis using widely accepted statistical methods and software. We have transparently outlined the specific statistical procedures and parameters used in the paper, providing a clear roadmap for others to replicate our statistical analyses.
Randomization	Participants were enrolled in two separate cohorts because of COVID-19 restrictions regarding personnel laboratory access, such that half enrolled in fall (September through November) 2020 and the other half in spring (March through May) 2021. Participants were matched for BW, BMI, and body fat and randomly assigned in parallel to one of two groups: 1) IF-P (n = 21); or 2) a heart-healthy daily caloric restriction diet (n = 20) for 8 weeks. Both groups consumed similar total weekly calories and expended similar amounts of energy expenditure throughout the 4 weeks.
Blinding	Blinding was not relevant to our study as the dietary interventions consisted of different foods.

Reporting for specific materials, systems and methods

We require information from authors about some types of materials, experimental systems and methods used in many studies. Here, indicate whether each material, system or method listed is relevant to your study. If you are not sure if a list item applies to your research, read the appropriate section before selecting a response.

Materials & experimental systems

n/a	Involved in the study	
☒	☐	Antibodies
☒	☐	Eukaryotic cell lines
☒	☐	Palaeontology and archaeology
☒	☐	Animals and other organisms
☐	☒	Clinical data
☒	☐	Dual use research of concern
☒	☐	Plants

Methods

n/a	Involved in the study	
☒	☐	ChIP-seq
☒	☐	Flow cytometry
☒	☐	MRI-based neuroimaging

Clinical data

Policy information about clinical studies

All manuscripts should comply with the ICMJE guidelines for publication of clinical research and a completed CONSORT checklist must be included with all submissions.

Clinical trial registration	NCT04327141
Study protocol	https://classic.clinicaltrials.gov/ct2/results?cond=&term=NCT04327141&cntry=&state=&city=&dist=
Data collection	Participants were enrolled in two separate cohorts because of COVID-19 restrictions regarding personnel laboratory access, such that half enrolled in fall (September through November) 2020 and the other half in spring (March through May) 2021. Participants were matched for BW, BMI, and body fat and randomly assigned in parallel to one of two groups: 1) IF-P (n = 21); or 2) a heart-healthy daily calorie restricted diet (n = 20) for 8 weeks.
Outcomes	We pre-defined primary and secondary outcomes based on previous literature, including prior studies from our laboratory. Those outcomes demonstrating the most likely influence of the dietary interventions were considered primary and those with secondary impact were treated as such. The methods are described in detail in the manuscript.

Plants

Seed stocks	No seed stocks were used in the study.
Novel plant genotypes	No novel plant genotypes were used in the study.
Authentication	N/A
